# Incidence and predictors of adverse outcomes in patients with rheumatic mitral stenosis following percutaneous balloon mitral valvuloplasty: a study from a tertiary center in Thailand

**DOI:** 10.1186/s12872-024-04067-8

**Published:** 2024-07-29

**Authors:** Kamonnart Songduang, Yodying Kaolawanich, Khemajira Karaketklang, Nithima Ratanasit

**Affiliations:** 1 Department of Medicine, Faculty of Medicine Siriraj Hospital, Mahidol University, Bangkok, 10700 Thailand; 2 Division of Cardiology, Department of Medicine, Faculty of Medicine Siriraj Hospital, Mahidol University, Bangkok, Thailand; 3 Her Majesty Cardiac Center, Faculty of Medicine Siriraj Hospital, Mahidol University, Bangkok, Thailand

**Keywords:** Adverse outcomes, Percutaneous balloon mitral valvuloplasty, Rheumatic heart disease, Mitral stenosis, Mitral valve

## Abstract

**Background:**

Rheumatic mitral stenosis (MS) remains a common and concerning health problem in Asia. Percutaneous balloon mitral valvuloplasty (PBMV) is the standard treatment for patients with symptomatic severe MS and favorable valve morphology. However, studies on the incidence and predictors of adverse cardiac outcomes following PBMV in Asia have been limited. This study aims to evaluate the incidence and predictors of adverse outcomes in patients with rheumatic MS following PBMV.

**Methods:**

A retrospective cohort study was conducted on patients with symptomatic severe MS who underwent successful PBMV between 2002 and 2020 at a tertiary academic institute in Thailand. Patients were followed up to assess adverse outcomes, defined as a composite of cardiac death, heart failure hospitalization, repeat PBMV, or mitral valve surgery. Univariable and multivariable analyses were performed to identify predictors of adverse outcomes. A p-value of < 0.05 was considered statistically significant.

**Results:**

A total of 379 patients were included in the study (mean age 43 ± 11 years, 80% female). During a median follow-up of 5.9 years (IQR 1.7–11.7), 74 patients (19.5%) experienced adverse outcomes, with an annualized event rate of 2.7%. Multivariable analysis showed that age (hazard ratio [HR] 1.03, 95% confidence interval [CI] 1.008–1.05, *p* = 0.006), significant tricuspid regurgitation (HR 2.17, 95% CI 1.33–3.56, *p* = 0.002), immediate post-PBMV mitral valve area (HR 0.39, 95% CI 0.25–0.64, *p* = 0.01), and immediate post-PBMV mitral regurgitation (HR 1.91, 95% CI 1.18–3.07, *p* = 0.008) were independent predictors of adverse outcomes.

**Conclusions:**

In patients with symptomatic severe rheumatic MS, the incidence of adverse outcomes following PBMV was 2.7% per year. Age, significant tricuspid regurgitation, immediate post-PBMV mitral valve area, and immediate post-PBMV mitral regurgitation were identified as independent predictors of these adverse outcomes.

## Introduction

Rheumatic heart disease, particularly mitral stenosis (MS), remains a notable health issue in Asia, including Thailand, leading to considerable mortality and morbidity, such as heart failure and cardiac death [1]. The management of MS has significantly evolved since the early 1980s with the introduction of the Inoue balloon [2]. Percutaneous balloon mitral valvuloplasty (PBMV) has become the intervention of choice for patients with symptomatic severe MS and favorable valve morphology [3, 4]. 

Previous retrospective cohort studies have evaluated the long-term outcomes of rheumatic MS patients undergoing PBMV, demonstrating an incidence of adverse cardiac outcomes ranging from 16 to 19% [5–9]. Predictive factors of adverse cardiac outcomes were identified, including New York Heart Association (NYHA) functional class, atrial fibrillation, and immediate post-PBMV mitral valve area [5–9]. 

However, limited data exist on the long-term adverse cardiac outcomes of rheumatic MS patients undergoing PBMV in Asia, including Thailand, where rheumatic heart disease is prevalent. Therefore, this study aims to evaluate the incidence and predictors of adverse cardiac outcomes in patients with rheumatic MS undergoing PBMV at a tertiary academic institution in Thailand.

## Methods

### Study population

This was a retrospective cohort study. Eligible patients were those aged 18 years or older with symptomatic severe rheumatic MS who underwent PBMV using the Inoue technique between July 2002 and September 2020 at Siriraj Hospital, Bangkok, Thailand. The diagnosis of severe MS was defined according to the guidelines at that time. In cases where patients underwent more than one PBMV procedure, only information from the first procedure during the study period was considered, with any subsequent procedures counted as outcomes. Only patients who had complete transthoracic echocardiographic data both before and immediately after the procedure were included. Patients with missing information on procedural success, missing echocardiographic data, or insufficient follow-up time were excluded from the study. The study was conducted in accordance with the Declaration of Helsinki. The institutional ethics committee (Siriraj Institutional Review Board [SIRB], Faculty of Medicine Siriraj Hospital, Mahidol University) approved this retrospective study and waived the need for additional written informed consent.

### Echocardiography

All patients underwent comprehensive echocardiography before and immediately after PBMV, in accordance with guideline recommendations [10]. The mitral valve area was measured using transthoracic echocardiography with 2-dimensional planimetry in the parasternal short-axis view. If planimetry was not available, the mitral valve area was determined using the pressure half-time method. Mitral valve morphology was assessed using the standard Wilkins echocardiographic scoring system, which involves semiquantitative grading of four components: leaflet mobility, valve thickening, subvalvular fibrosis, and valvular calcification [11]. Immediate post-PBMV transthoracic echocardiographic results were collected and included immediate post-PBMV mitral valve area, immediate post-PBMV mitral valve pressure gradient, and post-PBMV mitral regurgitation (MR). Right ventricular systolic pressure (RVSP) was estimated using tricuspid regurgitation (TR) maximum velocity and right atrial pressure. The severity of TR was assessed using multiple methods, such as visual assessment, vena contracta width/area, and regurgitant volume [12]. Significant TR was defined as moderate to severe TR by any assessment method.

### PBMV technique

The decision for the mitral valve intervention procedure was made by a heart team that considered clinical and echocardiographic data (e.g., age, comorbidities, surgical risk, severity of MR, presence of left atrial thrombus) as well as the mitral valve Wilkins score to decide whether each patient should undergo PBMV or mitral valve replacement. All PBMV procedures were performed using an Inoue balloon catheter via an anterograde trans-septal approach. Both right and left cardiac catheterizations were conducted before and during the procedure to evaluate hemodynamic alterations. The appropriate balloon size (in millimeters) was determined using the formula: (height in cm / 10) + 10. The balloon was gradually inflated from lower to higher volumes. Following each inflation, alterations in the transmitral mean pressure gradient and the extent of MR were monitored. Based on the interventionist’s judgment and in order to achieve optimal results, balloon inflation could be continued up to 1–2 mm more than the estimated size. In the final stage, left ventriculography was conducted to assess the degree of final MR.

### Clinical follow-up

Follow-up data were collected through clinical visits and medical records. Patients were monitored for adverse cardiac outcomes, which included a composite of cardiac death, heart failure hospitalization, repeat PBMV, or mitral valve surgery. Cardiac death was defined according to standard recommendations [13]. In cases where patients experienced multiple events, only the first event was considered for event-free survival analysis.

### Statistical analysis

Statistical analyses were performed using IBM SPSS Statistics for Windows, version 20.0 (IBM Corp., Armonk, NY, USA). Continuous variables with a normal distribution were reported as mean ± standard deviation (SD), while continuous variables with a non-normal distribution were reported as median and interquartile range (IQR). The normality of the variable distribution was assessed using the Kolmogorov-Smirnov test. Categorical variables were presented as absolute numbers and percentages. Differences between groups were compared using the Student’s unpaired t-test or Mann-Whitney U test for continuous variables, and the chi-square test or Fisher’s exact test for categorical variables, as appropriate. Event rates were estimated using the Kaplan-Meier method. To analyze predictors of composite adverse outcomes, we conducted a Cox regression analysis to assess univariable predictors based on baseline characteristics and echocardiographic variables. Variables with a p-value < 0.05 in the univariable analysis were included in the multivariable analysis to identify independent predictors. The results of the Cox regression analysis are presented as hazard ratios (HR) with corresponding 95% confidence intervals (CI). A p-value < 0.05 was considered statistically significant for all tests.

## Results

**Fig. 1:**
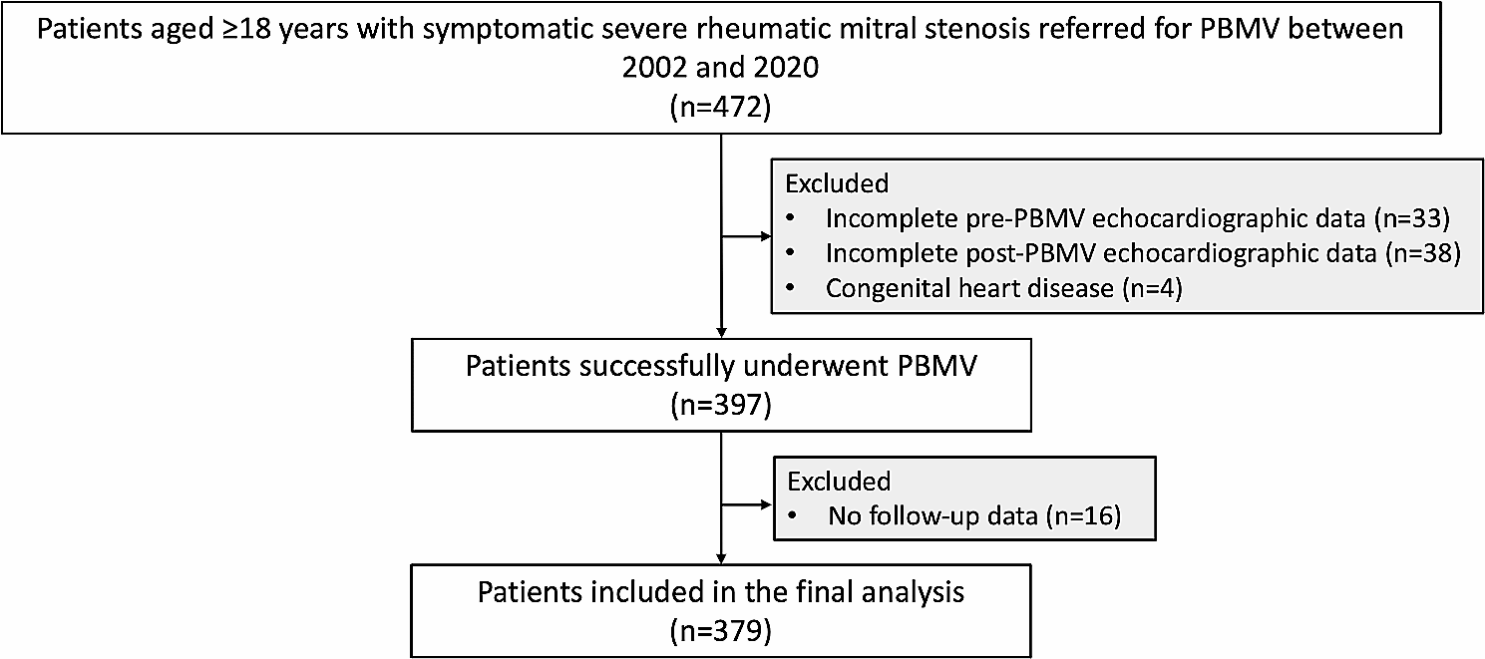
Study flow chart. Abbreviation: PBMV = percutaneous balloon mitral valvuloplasty

A total of 472 patients were studied. Ninety-three patients were excluded for the following reasons: incomplete pre-PBMV echocardiographic data in 33 patients, incomplete post-PBMV echocardiographic data in 38 patients, congenital heart disease in 4 patients, and incomplete follow-up data in 16 patients. Therefore, 379 patients were included in the final analysis. Figure 1 illustrates the patient flowchart. The median follow-up time was 5.9 years (IQR 1.7, 11.7). Adverse outcomes occurred in 74 (19.5%) patients, with an annualized event rate of 2.7%. Table 1 demonstrates the baseline characteristics, echocardiographic, and procedural data of the study population, with comparison between those with and without adverse outcomes.

**Table 1:** Baseline characteristics, echocardiographic, and procedural data of the study population, with comparison between those with and without adverse outcomes

Variables	Total	Adverse outcomes	No adverse outcomes	*P*-Value
	(*n* = 379)	(*n* = 74)	(*n* = 305)	
Age (years)	43.7 ± 11.4	45.9 ± 10.4	43.1 ± 11.6	0.06
Female gender	306 (80.7)	58 (78.4)	248 (81.3)	0.57
Atrial fibrillation	201 (53.0)	48 (64.9)	153 (50.2)	0.02
NYHA functional class III-IV	69 (8.2)	39 (52.7)	30 (9.8)	***< 0.001***
Medications				
Beta blocker	319 (84.2)	62 (83.8)	257 (84.3)	0.92
Penicillin	92 (24.3)	15 (20.3)	77 (25.2)	0.37
Diuretics	267 (70.4)	57 (77)	210 (68.9)	0.17
Digoxin	127 (33.5)	30 (40.5)	97 (31.8)	0.15
Pre-PBMV echocardiography				
Wilkin score, median (IQR)	8 (8, 9)	8 (8, 9)	8 (8, 9)	0.95
MVA (cm^2^)	0.92 ± 0.22	0.92 ± 0.21	0.92 ± 0.22	0.86
RVSP (mmHg)	50.2 ± 18.1	50.7 ± 17.7	50.1 ± 18.2	0.08
LVEF (%)	62.3 ± 8.9	63.2 ± 8.2	62.0 ± 9.1	0.31
LA dimension (mm)	55.2 ± 8.4	57.5 ± 7.4	54.7 ± 8.6	***0.01***
Significant TR	86 (22.6)	25 (33.8)	61 (20.0)	***0.01***
Procedural data				
Pre-procedural MV gradient (mmHg)	13.3 ± 4.7	12.7 ± 4.9	13.8 ± 4.5	0.23
Post-procedural MV gradient (mmHg)	6.4 ± 2.5	6.2 ± 2.4	6.8 ± 2.6	0.27
Pre-procedural systolic PAP (mmHg)	56.6 ± 18.3	59.0 ± 17.9	54.9 ± 19.2	0.29
Post-procedural systolic PAP (mmHg)	50.6 ± 15.4	53.7 ± 17.1	48.1 ± 13.5	0.08
Pre-procedural diastolic PAP (mmHg)	26.6 ± 9.8	26.4 ± 8.0	26.8 ± 11.0	0.85
Post-procedural diastolic PAP (mmHg)	22.9 ± 7.7	23.8 ± 6.6	22.2 ± 8.4	0.34
Pre-procedural mean PAP (mmHg)	39.2 ± 12.5	41.8 ± 12.5	37.4 ± 12.4	0.20
Post-procedural mean PAP (mmHg)	34.7 ± 9.5	39.8 ± 10.2	30.6 ± 6.4	***< 0.001***
Pre-procedural LA pressure (mmHg)	27.1 ± 7.0	26.0 ± 6.0	27.7 ± 7.6	0.47
Post-procedural LA pressure (mmHg)	22.2 ± 5.3	22.7 ± 6.1	21.9 ± 4.9	0.77
Immediate post-PBMV echocardiography				
IMVA (cm^2^)	1.91 ± 0.58	1.67 ± 0.65	1.97 ± 0.55	***< 0.001***
IMVPG (mmHg)	6.3 ± 2.7	7.2 ± 3.2	6.0 ± 2.6	***0.001***
IMR	74 (19.5)	31 (41.8)	43 (14.1)	***0.008***

Data are expressed as number (%), mean ± standard deviation, or median and IQR. Bold-italic values are < 0.05

Abbreviations: IMR = immediate post valvulotomy mitral regurgitation, IMVA = immediate post valvulotomy mitral valve area, IMVPG = immediate post valvulotomy mitral valve pressure gradient, IQR = interquartile range, LA = left atrium, LVEF = left ventricular ejection fraction, MV = mitral valve, MVA = mitral valve area, MR = mitral regurgitation, NYHA = New York Heart Association, PAP = pulmonary artery pressure, PBMV = percutaneous balloon mitral valvuloplasty, RVSP = right ventricular systolic pressure, TR = tricuspid regurgitation

The mean age was 43.7 ± 11.4 years, and 80.7% were women. Sixty-nine patients (8.2%) were in NYHA functional class III-IV, and 53% had atrial fibrillation. The mean mitral valve area was 0.92 ± 0.22 cm², and the median Wilkin score was 8 (IQR 8, 9). Patients with adverse outcomes had a higher proportion of those in NYHA functional class III-IV (52.7% versus 9.8%, *p* < 0.001), a greater left atrial atrial dimension (57.5 ± 7.4 versus 54.7 ± 8.6 mm, *p* = 0.01), and a higher prevalence of significant TR (33.8% versus 20.0%, *p* = 0.01) compared to those without adverse outcomes. There was no significant difference in mitral valve area and Wilkin score between patients with and without adverse outcomes. Patients with adverse outcomes had a significantly lower immediate post-PBMV mitral valve area, a higher immediate post-PBMV mitral valve pressure gradient, and a higher prevalence of immediate post-PBMV MR compared to those without adverse outcomes (all *p* < 0.05).

**Fig. 2:**
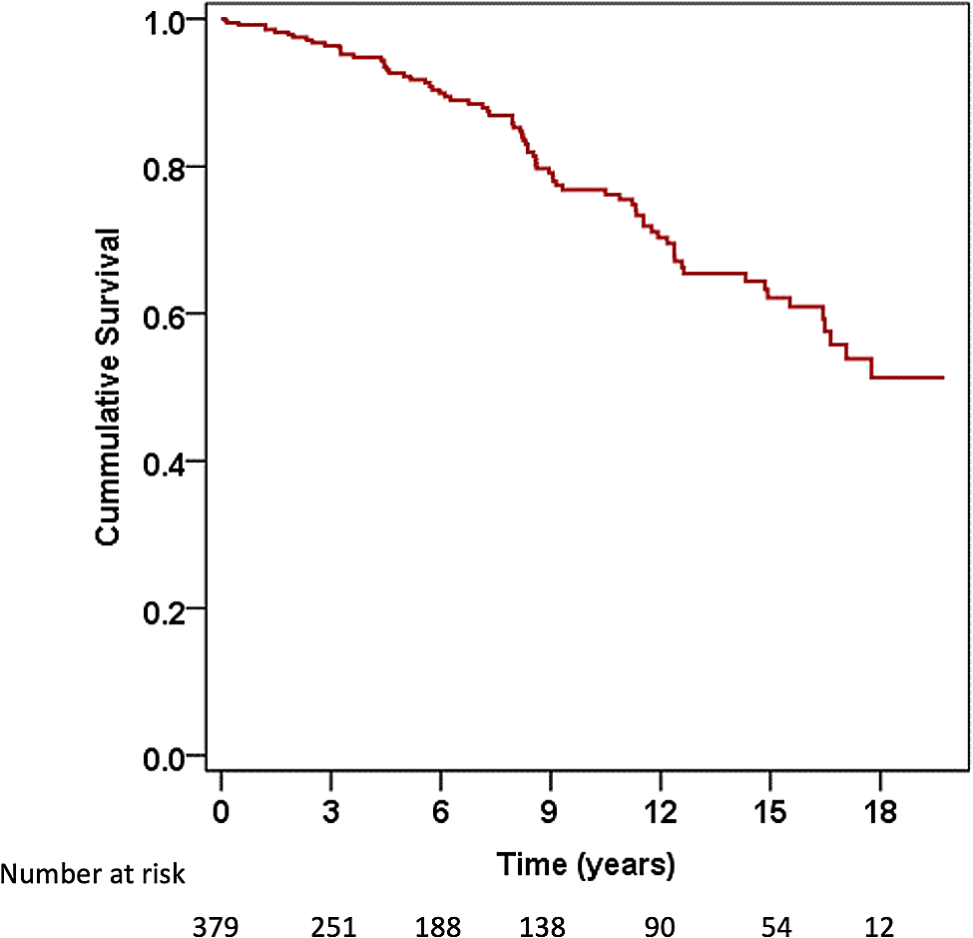
Kaplan-Meier survival curve of patients with rheumatic mitral stenosis undergoing percutaneous balloon mitral valvuloplasty

**Fig. 3:**
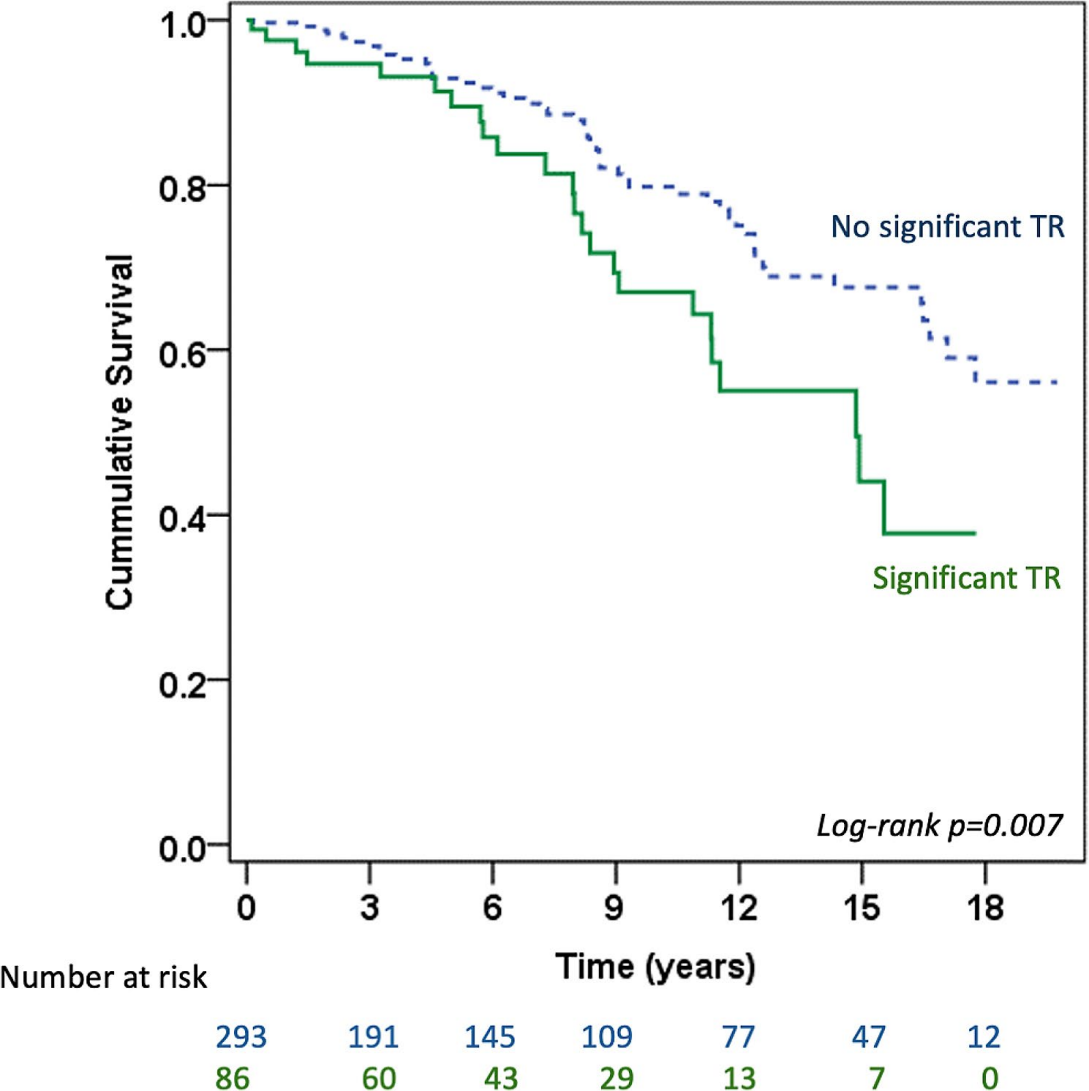
Kaplan-Meier survival curve of patients with rheumatic mitral stenosis undergoing percutaneous balloon mitral valvuloplasty comparing patients with and without significant TR. Abbreviation: TR = tricuspid regurgitation

Figure 2 shows the Kaplan-Meier survival curve of patients with rheumatic MS undergoing PBMV. Table 2 presents the numbers of each adverse outcome. Most adverse outcomes occurred due to a repeat PBMV or mitral valve surgery (66 patients; 17.4%). Table 3 presents the univariable and multivariable Cox regression analyses for the predictors of adverse outcomes. Univariable analysis revealed that age, atrial fibrillation, NYHA functional class, significant TR, immediate post-PBMV mitral valve area, post-PBMV mitral valve pressure gradient, and immediate post-PBMV MR were associated with adverse outcomes (all *p* < 0.05). Multivariable analysis identified age (HR 1.03, 95% CI 1.008–1.05, *p* = 0.006), significant TR (HR 2.17, 95% CI 1.32–3.56, *p* = 0.002), immediate post-PBMV mitral valve area (HR 0.39, 95% CI 0.25–0.64, *p* = 0.009), and immediate post-PBMV MR (HR 1.91, 95% CI 1.18–3.07, *p* = 0.008) as independent predictors of adverse outcomes. Figure 3 shows the Kaplan-Meier survival curve of patients with rheumatic MS undergoing PBMV, comparing those with and without significant TR. Patients with significant TR had a significantly higher rate of adverse outcomes than those without significant TR (log-rank *p* = 0.007).

**Table 2:** Adverse cardiac outcomes

	Total
	(*n* = 379)
Composite outcomes	74 (19.5)
Cardiac death	4 (1.1)
Hospitalization for heart failure	3 (0.8)
Repeat PBMV	3 (0.8)
Mitral valve surgery	66 (17.4)

Data are expressed as number (%)

Abbreviations: PBMV = percutaneous balloon mitral valvuloplasty

**Table 3:** Univariable and multivariable Cox regression analyses for the predictors of adverse cardiac outcomes

Variables	Univariable HR (95% CI)	Multivariable HR (95% CI)	*P*-value
Age (years)	1.02 (1.005–1.05)	1.03 (1.008–1.05)	***0.006***
Atrial fibrillation	1.92 (1.19–3.09)		
NYHA functional class III-IV	6.18 (3.90–9.80)		
Significant TR	1.93 (1.19–3.13)	2.17 (1.32–3.56)	***0.002***
IMVA (cm^2^)	0.43 (0.34–0.76)	0.39 (0.25–0.64)	***0.009***
IMVPG (mmHg)	1.11 (1.005–1.22)		
IMR	2.05 (1.29–3.26)	1.91 (1.18–3.07)	***0.008***

Bold-italic values are < 0.05

Abbreviations: CI = confidence interval, HR = hazard ration, IMR = immediate post valvulotomy mitral regurgitation, IMVA = immediate post valvulotomy mitral valve area, IMVPG = immediate post valvulotomy mitral valve pressure gradient, NYHA = New York Heart Association, TR = tricuspid regurgitation

## Discussion

The main findings of the study were that during the median follow-up time of 5.9 years, 19.5% of patients with rheumatic MS undergoing PBMV experienced adverse cardiac outcomes, with an annualized event rate of 2.7%. Age, significant TR, immediate post-PBMV mitral valve area, and immediate post-PBMV MR were identified as independent predictors of adverse outcomes, with significant TR emerging as the strongest predictor.

Rheumatic heart disease remains a significant health concern in many parts of the world, particularly in low- and middle-income countries, where it is one of the leading causes of cardiovascular morbidity and mortality [14]. Ou et al. reported global trends in rheumatic heart disease, noting increasing trends in the age-standardized rates of incidence and prevalence worldwide. The respective estimated annual percentage changes were 0.58 and 0.57, with increasing trends commonly observed in low- and middle-socioeconomic countries [15]. 

PBMV should be considered as an initial treatment for selected patients who exhibit mild to moderate calcification or impaired subvalvular apparatus, but otherwise possess favorable clinical characteristics [16]. A recent meta-analysis showed lower procedural morbidity associated with PBMV compared with mitral valve replacement, thus supporting the recommendation of PBMV in young patients with suitable anatomy [17]. Several studies report long-term outcomes of patients with MS following PBMV, demonstrating an incidence of adverse outcomes ranging from 16 to 19% [5–9]. In our study, the incidence of adverse outcomes was comparable to prior studies, with a rate of 19.5%. Patients with adverse outcomes had a worse functional class and a greater left atrial dimension, which were also similar to previous studies [8, 9]. However, in our study, NYHA functional class was only a predictive factor in the univariable analysis. It should be noted that NYHA functional class, although a strong predictive factor for adverse outcomes, is a subjective variable. The clinical data from patients complaining of a defined NYHA functional class were assessed by physicians, which may result in differences in interpretation among patients.

Significant TR was the strongest predictor of adverse outcomes in our study, which was reported in prior studies. TR is most often the consequence of left-sided cardiac diseases that induce right-sided chamber dilatation, and hemodynamically significant TR can cause significant morbidity and mortality [18, 19]. Although rheumatic TR can occur, secondary TR due to pulmonary hypertension is far more common in patients with rheumatic heart disease. Significant TR can develop over time even after successful PBMV [20]. Sagie et al. studied the association between the presence of TR and immediate and late adverse outcomes in patients undergoing PBMV. They found that the prevalence of significant TR was 31%, and patients undergoing PBMV with significant TR exhibited advanced mitral valve and pulmonary vascular disease, suboptimal immediate results, and poor late outcomes [21]. Another study by Caldas et al. also showed that the prevalence of significant TR was 12.8% in patients with rheumatic MS undergoing PBMV and was independently associated with adverse outcomes [22]. Our study revealed a similar prevalence of significant TR to that reported by Sagie et al., but higher than that reported by Caldas et al. This difference could be attributed to variations in the definition of severity and the assessment methods used in the studies. Nevertheless, significant TR consistently emerges as a strong predictor of adverse outcomes in all studies, including ours.

Immediate post-PBMV mitral valve area has been associated with long-term outcomes in patients with rheumatic MS undergoing PBMV in prior studies [23, 24]. Our results also showed consistent findings. Significant MR following PMMV is a frequent event, mainly related to commissural splitting, with favorable clinical outcomes [25]. However, patients with damaged central leaflet scallop or subvalvular apparatus had the worst outcomes compared to patients with mild or commissural MR [26]. In our study, although we were unable to classify the severity or mechanism of immediate MR following PBMV, immediate post-PBMV MR still emerged as an independent predictor of adverse outcomes.

The clinical implication of our study is that PBMV in patients with severe MS demonstrated good long-term outcomes with a relatively low rate of adverse outcomes. Most adverse outcomes were mitral valve surgeries, with very low rates of mortality or heart failure. Additionally, our study highlighted significant known predictors of adverse outcomes, such as age, immediate post-PBMV mitral valve area, and immediate post-PBMV MR, as well as an emerging predictor, significant TR, which should be integrated into the care of this patient population.

### Limitations

Our study had several limitations. Firstly, the study methodology was retrospective, and therefore, some confounding factors could not be totally eliminated. However, multivariable analysis was performed to the best of our ability. Secondly, the PBMV procedures were performed by experienced operators in a tertiary center, which may limit generalization. However, the rate of adverse outcomes in our study was comparable to prior studies. Thirdly, we were unable to conduct follow-up echocardiography after discharge, which could be associated with adverse long-term outcomes. Fourthly, we defined the severity of other valvular functions rather than MS using multiple methods, including qualitative and quantitative methods, and were unable to quantify the severity of other valvular functions (e.g., TR) in every patient, which may not be consistent.

## Conclusion

In patients with symptomatic severe rheumatic MS, the incidence of adverse outcomes following PBMV was 2.7% per year. Age, significant TR, immediate post-PBMV mitral valve area, and immediate post-PBMV MR were identified as independent predictors of these adverse outcomes.

## Data Availability

The datasets used and/or analyzed during the current study are available from the corresponding author on reasonable request.

## Abbreviations

AF: Atrial fibrillation
CI: Confidence interval
HR: Hazard ratio
IQR: Interquartile range
MR: Mitral regurgitation
MS: Mitral stenosis
NYHA: New York Heart Association
PBMV: Percutaneous balloon mitral valvuloplasty
RVSP: Right ventricular systolic pressure
SD: Standard deviation
TR: Tricuspid regurgitation
